# ‘It was like a little family’: A qualitative study of online hearing voices peer support groups in the NHS

**DOI:** 10.1111/papt.70064

**Published:** 2026-04-12

**Authors:** Alison Branitsky, Samantha Bowe, Sandra Bucci, Anthony P. Morrison, Eleanor Longden, Lee D. Mulligan, Filippo Varese

**Affiliations:** ^1^ Division of Psychology and Mental Health, School of Health Sciences, Faculty of Biology, Medicine and Health, Manchester Academic Health Science Centre The University of Manchester Manchester UK; ^2^ Psychosis Research Unit, Greater Manchester Mental Health NHS Foundation Trust Manchester UK; ^3^ Complex Trauma and Resilience Research Unit Greater Manchester Mental Health NHS Foundation Trust Manchester UK

**Keywords:** hearing voices network, online support, peer support, qualitative study, voice hearing

## Abstract

**Objectives:**

To investigate the experiences of individuals attending an online hearing voices peer support group (HVG) conducted within the UK's National Health Service (NHS). The purpose of the study was to assess acceptability of the HVG in an NHS context and to understand relational dynamics within the group.

**Methods:**

A nested qualitative study was conducted within a non‐randomised feasibility trial of an online HVG. All participants (*N* = 9) completed baseline and end‐of‐study qualitative interviews about their voice hearing and group experiences. Interviews were analysed using framework analysis.

**Results:**

Data were organised into four themes. Participants described two orientation tendencies within the group, both with associated subthemes: (1) seeking connection *(shame and isolation spurred interest in the group*, *active digital participation and engagement facilitated a sense of connection*, *value of an alternative*); and (2) seeking to learn (*searching for solutions*, *cameras enabled the modulation of engagement but inhibited group ownership*, *a useful addition to care*). Participants likewise described (3) voices' experiences in the group; and (4) the impact of the group (*impact on voices*, *impact on sense of self and hope for the future*).

**Conclusions:**

Despite their differing expectations for the group, experiences in the group and relationship with the online medium of the group, both the participants seeking connection and those seeking to learn reported benefits from group attendance in terms of self‐acceptance and hope for the future. Further research is needed to understand how to incorporate this survivor‐led approach into the NHS.

## INTRODUCTION

Supporting the implementation of peer support into the UK's National Health Service (NHS) has been a priority over the past several years (NHS England, 2019). Peer support is premised on the idea that those with lived experience of mental distress are uniquely positioned to offer support to others (Davidson et al., 2006) and is associated with a range of positive outcomes, including increased social connectedness, hope and quality of life (Castelein et al., 2008; Fuhr et al., 2014). Peer support has a strong legacy in the voluntary sector and espouses values such as mutual responsibility, reciprocal relationships and collective ownership of peer spaces (Mead & MacNeil, 2006), which often stand in stark contrast to the ethos and practices of statutory services. Accordingly, concerns around the ‘over‐professionalisation’ of peer support in public mental health services (Adams, 2020; Otte et al., 2020; Simpson et al., 2018; Voronka, 2019), and the subsequent dilution of peer support's distinct values and qualities, has been raised (Gillard et al., 2024). Specifically, this has long been a concern of the Hearing Voices Movement (HVM), an international coalition between voice hearers and their allies, which has at times had an ‘uneasy relationship’ with traditional mental health services and scientific inquiry (Corstens et al., 2014, p. S289). One of the central prerogatives of the HVM is to support the establishment of Hearing Voices Groups (HVGs), peer support groups in which voice hearers can share their experiences openly and in‐depth without fear of consequences and without pressure to change (Hornstein et al., 2020). HVGs prioritise *relationships* over interventions and outcomes, and posit that building genuine, mutually responsible, reciprocal relationships can serve as a catalyst for subjectively defined psychological change (Hornstein et al., 2020, 2021). As such, the peer values of mutual responsibility, reciprocal relationships and collective group ownership are of central importance in HVGs; however limited research currently exists exploring the extent to which these values may be realised in NHS settings.

Since their inception in the 1980s, HVGs have been established in over 25 countries, with 50 groups currently operating in the voluntary sector in the UK (English Hearing Voices Network, 2024). Participants report numerous benefits from HVGs, including normalisation of voice hearing, enhanced coping with and understanding of voices, decreased isolation and hope for the future (Corentin et al., 2023; Kalofonos et al., 2023). Crucially, participants report that HVGs represent a unique form of support, particularly in terms of their style of interaction (Hornstein et al., 2020), indicating that the experiences in the group and the benefits garnered from attendance are distinct from those of other clinical services and interventions (Branitsky, Bowe, Morrison, et al., 2025; Hornstein et al., 2024; Longden et al., 2018). Therefore, given that many distressed voice hearers are referred to clinical services (Johns et al., 2014), there is utility in investigating whether HVG support is acceptable and beneficial to individuals who may be unable or unwilling to access groups outside the NHS.

Prior to the COVID‐19 pandemic only two HVGs were conducted remotely using digital communication platforms (Hearing Voices Network – USA, 2020); although, to date, more than 10 now operate in the UK alone (English Hearing Voices Network, 2024). Given the resource scarcity in the NHS, online groups may in turn offer an accessible and cost‐efficient form of support. However, previous research has highlighted some of the necessary considerations for successfully facilitating groups online (Branitsky, Longden, Bucci, et al., 2024) as the remote context may make establishing mutually responsible groups online more challenging (Weinberg, 2021).

Taken together, the ideological and contextual factors which may impede the implementation of online HVGs within the NHS mean further qualitative inquiries into these types of groups are warranted. As such, the current paper provides an extended exploration of the feasibility and acceptability of conducting an online HVG within NHS services to more fully understand participants' experiences of the group, the relational dynamics therein, and any reported impacts of group participation.

## METHOD

### Design

This qualitative study was conducted as part of a single‐site non‐randomised trial to evaluate the feasibility and acceptability of delivering an online HVG for NHS service users. All study participants were interviewed at baseline (*N* = 9) and invited to take part in a second interview at the end of the study (*N* = 8). The online HVG was delivered on a digital communication platform (Zoom) over the course of 6 months, was unstructured in nature without a pre‐set agenda, adhered to the values of peer support that underpin HVGs (Branitsky, Longden, Morrison, et al., 2024), and broadly focused on an exploration of attendees' voice hearing experiences. Additional information about group structure can be found elsewhere (Branitsky, Bowe, & Mulligan, 2025; Branitsky, Longden, Morrison, et al., 2024).

Topic guides were developed by AB in conjunction with patient and public involvement and engagement (PPIE) experts (Materials S1 and S2). In brief, baseline interviews focused on individuals' expectations of the group, including any subjective goals of attendance; hesitations about attendance; reflections on the online medium; and understanding participants' voice hearing experiences, including history, phenomenology, interpretation and impact of voices. End‐of‐study interviews broadly focused on the acceptability of group and trial structure, particularly as it pertained to the online medium and experiences within the group, including the extent to which the group met expectations and subjective goals, and the impact of the group on voices, sense of self, and life beyond the group. All questions were piloted with PPIE experts and topic guides were iterative, with questions being added or emphasised based on emergent findings. Reflective logs were maintained throughout data collection and analysis and informed the interpretation of the data. The Consolidated Criteria for Reporting Qualitative Research were additionally consulted in reporting the findings (Tong et al., 2007).

### Participants

Participants were recruited via secondary NHS mental health services, third‐party and voluntary sector organisations and could self‐refer via social media. Individuals were eligible to take part in the trial if they were: (1) aged 18 or older; (2) currently lived in the UK; (3) had heard voices for at least the past 6 months; (4) had sufficient access to the internet and ability to use videoconferencing platforms; (5) were willing to participate in group support; and (6) had the ability to provide informed consent. Participant pseudonyms and characteristics are presented in Table 1. All participants in the trial were invited to take part in the qualitative study.

**TABLE 1:** Participant characteristics.

Name	Age	Ethnicity	Service	Duration of voice hearing	Number of group meetings attended
Roger	19	White British	Early detection and intervention	7 years	21
Nigel	27	White British	Early intervention	2 years	23
Tyler	19	Mixed race	Early intervention; transitioned to community mental health team	3 years	8
Reese	30	White British	Early intervention; transitioned to community mental health team	3 years	18
Malcolm	58	White British	Community mental health team	44 years	11
Maggie	18	White British	Early intervention	1 year	5
Sofia	37	Other White background	Community mental health team	13 years	12
Angela	46	Asian	Early intervention	1 year	13
Rita	57	White British	Community mental health team	31 years	8

### Procedure

Study procedures were approved by the NHS West Midlands—Black Country Research Ethics Committee (23/WM/0045) and all participants provided written informed consent. Baseline and follow‐up qualitative interviews were conducted as part of the respective baseline and end‐of‐study assessments (see Branitsky, Longden, Morrison, et al., 2024) between May and October 2023 and March and May 2024. Interviews were conducted by AB, a lived‐experience researcher who also acted as the HVG's primary facilitator. Several steps were taken to reduce the possibility of a social desirability bias, including assuring participants that sharing negative experiences in the group was welcome and useful, reminding participants of times in the group where the participants and facilitator navigated difficult experiences, and reiterating that content shared during interviews would not affect future rights or care. All interviews were conducted online via Zoom (Zoom Video Communications Inc, 2023), audio recorded and transcribed verbatim, after which participants were assigned a pseudonym, and all identifiable information was removed. The mean interview length was 22:53 min (range: 09:13–48:27) for baseline interviews and 48:34 (range: 25:27–87:50) for end‐of‐study interviews. Participants were compensated £20 at each assessment.

Questions about expectations of the group and sense of self were asked at both baseline and end‐of‐study and responses relevant to these topics were retained for the analysis. However, the remainder of data from the baseline interviews was not included as it did not yield sufficient information as to enable a longitudinal analysis. A substantial part of the baseline qualitative interviews centred on voice phenomenology, and most participants were, at the time of the interview, unable or unwilling to discuss voice phenomenology, which precluded a phenomenological comparison between baseline and end‐of‐study (see Materials S1 and S2 for topic guides).

### Analysis

The analysis adopted a critical realist epistemological framework. Data were analysed using Framework Analysis (FA; Ritchie & Spencer, 1994), a methodology that fits within the class of ‘thematic’ approaches, provides a structured approach to data analysis, and is suitable for research, such as feasibility and acceptability studies, where acceptability is a specific focus (Midgley et al., 2015; Parkinson et al., 2016). As such, FA was chosen because it enables harmonising a priori areas of interest with emergent findings when developing an analytic framework (Parkinson et al., 2016). As a qualitative study nested within a feasibility and acceptability trial, several issues pertaining to acceptability were prioritised, including participants' experiences of relational dynamics in the group, although the nuances of individuals' subjective experiences were likewise included.

The analysis was conducted according to the recommendations of Ritchie and Spencer (1994) and adhered to the following process: (1) data familiarisation through repeated reading of interview transcripts; (2) developing a framework through which to organise the data; (3) indexing the data by coding each meaningful piece of text into one or more categories from the framework; (4) charting the data by summarising the data in each category for each participant; and (5) mapping and interpreting the data. The framework used in stage two was developed a priori to answer specific questions about acceptability and contained the following: (1) reasons for coming to the group; (2) group practicalities (e.g., structure, cameras, facilitator); (3) connection and disconnection in the group; (4) impact and change; and (5) value of an alternative. The final interpretation was informed both by the a priori framework, themes that emerged from the data, and AB's reflections as the facilitator of the HVG. The initial framework and final interpretation were derived by AB in consultation with EL, SBu, APM and FV. Data analysis took place using NVivo (version 12; Lumivero, 2024). As FA emphasises the researcher's active role in data generation and knowledge production (Yardley, 2008), we did not seek to establish interrater reliability. Validation of the final analysis was sought from study participants, all of whom consented to member checking, however this offer was not taken up by any participant. The analysis was likewise validated by PPIE representatives, who suggested no substantial changes to the thematic structure.

Traditionally, the experiences and opinions of the voices themselves have not been included in research on psychological interventions. However, many individuals hear voices which are sufficiently personified to hold and express their own opinions on the world around them, and PPIE experts have noted that the experiences and opinions of voices should be enquired about and included in research. Given that HVGs are premised on understanding and acceptance for voices, it is important to consider how voices themselves find taking part in groups. While voices were not asked directly about their experiences in the group, participants were asked to indirectly report on voices' experiences in the group and consideration was given in the interviews to understanding the perspectives of voices and ensuring that their reflections were included in the analysis.

### Reflexivity and situating the group

The researchers in this study hold perspectives on voice hearing that align with the ethos of the HVM. AB is a PhD‐level researcher, EL is an academic psychologist, and SBo, SBu, APM, LDM and FV are academic clinical psychologists. AB is trained in HVG facilitation and Intentional Peer Support (Mead, 2010; Mead et al., 2013; Mead & MacNeil, 2005), and strove to bring the ethos of those approaches, particularly that of fostering mutual, reciprocal relationships, to the current group. AB has experience facilitating face‐to‐face and online groups in the United States that took place in peer support communities. She likewise has facilitated community‐based HVGs in the UK, as well as attended groups in the community that are facilitated by NHS employees. AB, SBo and LDM facilitated the online HVG: AB in her capacity as a lived‐experience researcher and group facilitator, and SBo and LDM in their capacity as clinical psychologists. Reflective logs kept by AB during the study were intentionally used to interpret and contextualise qualitative findings. We did not seek objectivity in terms of the results; rather, we sought to use the research team's perspectives to further nuance and legitimise the findings.

To appropriately contextualise the results, the current HVG should be understood within the larger HVG landscape. As this group took place as part of research within the NHS, it was bound to NHS governance requirements, such as note taking and risk reporting (see Branitsky, Longden, Morrison, et al., 2024). As a result, from the perspective of facilitators, it made the peer values of mutual responsibility, reciprocal relationships and collective group ownership difficult to fully realise (for a full discussion, see Branitsky, Bowe, & Mulligan, 2025). This was further compounded by the remote nature of the group. Consequently, the results presented below should be understood as inherently context‐bound and therefore may not represent the experiences of HVG participants in other group settings.

## RESULTS

### Results are organised into four main themes and associated subthemes

**FIGURE 1:**
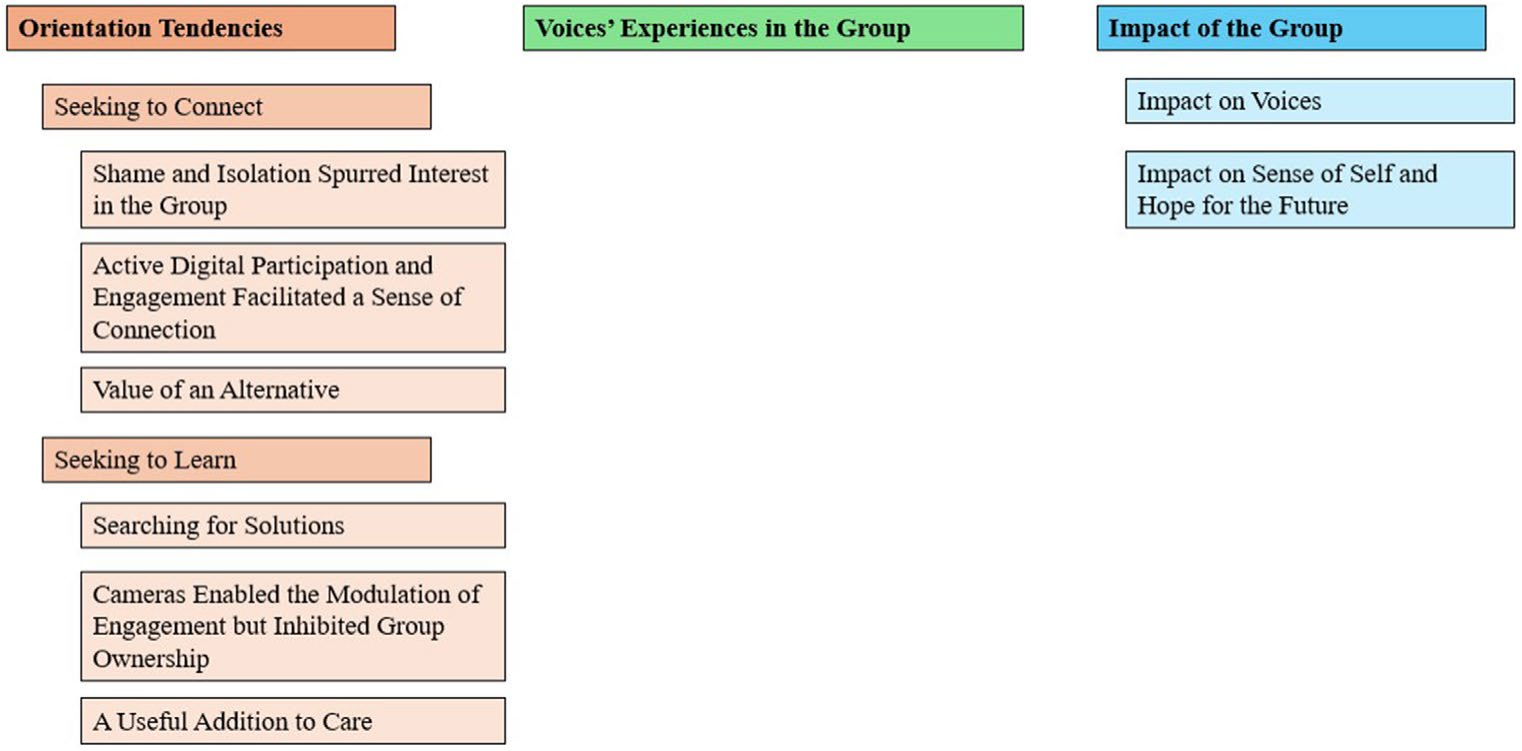
Thematic map.

The first two themes outline distinct relational orientation tendencies described by group participants: *seeking connection* and *seeking to learn*. The subthemes delineate these participants' reasons for attending, their experiences in the group, and how the online medium impacted their relationship with other group members. These orientation tendencies should be understood as porous and non‐prescriptive, with several participants transitioning from seeking to learn to seeking to connect over the course of the group. The second two themes: *voices' experiences in the group* and *impact of the group* describe experiences that existed across the two relational patterns. A thematic map is presented in Figure 1.

### Seeking connection

Several participants described a relational orientation tendency that was centred around connecting with other group members. These individuals were primarily focused on understanding and being understood by others. Feelings of shame and rejection were also common for them; as such, the primary goal of connecting was to foster a sense of shared humanity and move away from the isolation that had plagued them for years. For these participants, the group was a space of active collaboration and co‐creation, rather than a place to passively absorb information. In terms of characteristics, these participants tended to be older, had a longer duration of voice hearing, and had more long‐standing involvement with mental health services. By extension, they additionally tended to have more experience with being in group settings, and therefore were more familiar with the necessary give‐and‐take required for groups to flow.

#### Shame and isolation spurred interest in the group

Participants reported numerous reasons for attending the group, many of which centred on a desire to meet and connect with other people who had similar experiences. For some participants, this was because they never had the opportunity to meet other voice hearers outside of a hospital setting:

I don't think I've ever really talked to anybody. I've seen people in hospitals that are voice hearers. […]But I've never spoken to anybody about – I don't know anybody, outside of being, you know, so unwell in hospital, that hears voices. So, I've never spoken to anybody about it. (Rita)

Only seeing other voice hearers in a hospital context reinforced harmful ideas that voice hearers needed to be frequently confined and could not live normal lives outside of restrictive settings. As such, these participations were left feeling ‘*crazy*’ (Sofia) and often held a deep sense of ‘*isolation*’ (Reese), and ‘*shame*’ (Rita) around hearing voices.

Participants had often experienced stigma and discrimination in the past, and when they had tried to discuss their voices, they were met with an insistence that they ‘*top up [their] medication*’ (Rita). As such, they were keen to find a compassionate space in which to discuss their voices without fear of consequences and, therefore, be afforded the opportunity to explore and understand their experiences more fully.

The element of peer support was particularly appealing to these participants. There was a hope that other voice hearers might hold unique knowledge that could enhance one's own understanding of the voices' origin and nature. As Sofia described, ‘*I just always wanted to know and find out if other people get the same voices as I do. And why do they get them’*.

While those who came to the group seeking connection expressed a desire to hear others' stories, they also recognised the importance of being able to share their own experiences. As Reese described, ‘*I don't think I've got any goals, it's more like being able to talk about my experiences really’*. It was hoped that others would be able to resonate with their stories, providing a sense of camaraderie to combat overwhelming feelings of shame and isolation.

These participants likewise recognised that the group was a co‐created space that would be actively shaped by their contributions. As Rita expressed it, ‘*I think I'll get out of it what I put into it*’. Similarly, the importance of sharing also reflected a desire to be seen and heard by other people:

I have no delusion that I'm gonna come to this group and my voices are gonna be cured. It's nothing like that… It's really, really powerful to share. And just know that others know. Just know that others know and, and get you…and I've never had that with my voice hearing… To be able to share is important to me. For the first time ever, to be able to share my voice hearing experience. (Rita)

This desire to be witnessed endowed participants with a sense of responsibility: to bear witness to the stories of others. This manifested in the ways those seeking to connect acted in the group: they had their cameras on, asked questions of other group members, reflected on the experiences of others and how it mapped onto their own experiences, and extended words of compassion and empathy towards participants in distress.

Nevertheless, these participants could also express understandable hesitation about joining the group. This tended to stem both from fear that the group would be emblematic of past negative experiences in hospital (‘*[I was] a bit apprehensive at first*…*cos I've never done anything like that before and the first [HVG] I did when I was in hospital didn't go so well*’ [Reese]) and from concerns that their experiences would be seen as more ‘*extreme*’ (Rita) than the other group members'. Both hesitations were rooted in a fear of continuing to be isolated and seen as ‘*different*’ (Reese) from others.

#### Active digital participation and engagement facilitated a sense of connection

As the group convened via Zoom, participants had a choice about whether to turn their cameras on or keep them off. For participants seeking connection, being able to see other group members was crucial to their sense of belonging to the group as they could verify whether others were engaged and empathic to the topic being discussed and the emotions that underpinned the narrative (‘*you can see the emotions behind the story*’: Tyler). As such, having cameras on was seen as an expectation for mutual relationships, and intentions and engagement could not be adequately discerned in the absence of visual information.

Some participants came into the group with the expectation that everyone would be on camera and were subsequently disappointed when other group members were reticent to be seen as this inhibited the construction of reciprocal relationships. In part, this could also be seen as a function of generational norms around technology use:

I gather now from my young son, people attend meetings and stuff at work with their cameras off and stuff. Now I didn't know that was a thing. So I found, I thought it was quite rude at first. But then I understood it more that that's the way people are online these days. (Rita)

For many participants, having their camera on was a direct expression of their confidence, both in their ability to interact with the group and in their comfort as being seen and identified by others as a voice hearer. This is perhaps paradoxical, given that these participants were the same ones who reported shame and embarrassment over being a voice hearer, but as Sofia stated, ‘*I think I'm maybe more confident in myself and I'm not scared to show the way I am’*. Malcolm explicitly wanted to be a model for the younger group members: ‘I *think it's important [to have your camera on]*…*for me on a personal level, I just wanted to show people that*…*living with voices in your head, you know, it isn't all bad’*. Having cameras on may perhaps also be seen as a more direct way of combatting the isolation that many of these participants reported.

Many participants felt uneasy and uncomfortable when they were the only one with their camera on as it reinforced a sense of isolation and difference. At times, this acted as a source of disconnection:

I wasn't happy that I was the only one apart from [Malcolm] that turned their camera on at the beginning. But then [Malcolm] didn't attend some weeks when I attended. It was literally you, me, and [facilitator] were the only ones with the camera on. I didn't like that. I felt quite conspicuous…I think when they were all off, I didn't feel part of the group. I felt very exposed. And as if I was the only one there. And people were looking in on me and I couldn't see them. (Rita)

In this respect, feeling watched, alone and different from others replicated the precise feelings that group members were hoping that their participation would alleviate. A turning point for the group came when one member explicitly asked why others did not have their cameras on and stated that she felt isolated in the group:

It was really a bit annoying…because I felt like I'm just talking with myself, looking at myself while talking…after a couple of sessions you start to lose interest…like you feel like nobody's taking it seriously…I just came to the point where I [was] kind of fed up and wanted to know why they didn't turn on the cameras and I just asked. (Sofia)

While not every group member turned their camera on, some, like Reese, did as an act of ‘*solidarity*’ indicating that these participants felt a responsibility to respond empathically to the needs of other members, even if it caused some level of personal anxiety. Furthermore, for participants like Reese (who initially reported immense hesitation around being seen) it was responding to the needs of others that enabled him to push himself to turn his camera on and participate in a way that he doubted was possible for him, a decision he ultimately felt immensely proud of.

Participants reported being able to ‘*connect a bit more*’ (Sofia) to group members who had their cameras on, as seeing others enabled the development of trust, the discernment of intent, and feelings of authenticity, all of which ultimately enabled participants to feel less alone. Even for participants like Nigel, who was primarily seeking to learn, the nonverbal cues available when cameras were on enhanced the group experience: ‘*I think it was good to see their face, their voice. It felt like, able to connect more easily when they were showing their faces’*. In turn, members felt they were able to share more intimately when others had their cameras on: ‘*I think I was able to share more when their cameras were on. Like I can more trust if you know what I mean*’ (Nigel), as participants could tell that other members were listening, engaged, and moved by what was being shared. For Rita, being seen was a central part of having her experiences be adequately witnessed, as it directly combatted the shame she had previously felt, ‘*to me it was important that people see me. I was sharing, you know, quite intimate stuff sometimes. And I think it was important for me that people see me as a person and not just a voice’*.

#### Value of an alternative

For many participants, hearing and reflecting on others' stories enabled them to gain deeper insight into their own voices by juxtaposing them with the experiences of others. For some, this enabled them to appreciate, for the first time, ‘*how far I've actually come*’ (Tyler). As Malcolm described:

I think I surprised myself sometimes and thinking, I've not got it under control – the voices in my head…but I feel like I've got that little bit of control over it…I mean the guys in the Zoom room, I could tell that they struggle more than me…I suppose it's years of trying to accept the voices.

Other participants were able to gain specific knowledge about experiences that had long been troubling them, which in turn facilitated a new level of self‐acceptance:

I asked you like…do you think [there's] an explanation for why I'm hearing… ‘I'm gonna kill my best friend’ and you said for protection or other things and it was really helpful to know that I wasn't completely out of my mind. There is an explanation to that. (Sofia)

Participants who came to the group seeking connection placed particular value on the group being a user‐informed alternative to traditional mental health support with distinct values and ethos. Specifically, participants appreciated the emphasis on autonomy, self‐determination, and expertise by experience. As Reese stated, ‘*we'd never really heard that saying before, about being the own expert*’, which in turn allowed him to speak about his experiences more authentically without fear of consequences:

Being able to talk about my voices openly and stuff. And like not being afraid of saying, ‘oh yeah I've struggled this week’ without being told ‘right you have to do this and this.’ You can say, ‘I've struggled this week’ and then hear other people saying how they coped with it when they had struggles similar to myself.

Rita emphasised that the ‘*message*’ of the group was distinct, and that she was treated as though she had a worthwhile contribution:

I felt very different. And treated differently. I was just a normal person in the group. You know, giving my contributions and sharing and getting stuff back from other people sharing. Whereas the message I get from the team is always ‘you're ill and you need to take this medication.’ That's always the message. And it's not always helpful.

For these participants, having a group facilitator with lived experience of voice hearing was ‘*very important*’ (Sofia), as mental health professionals ‘*can't understand us a hundred percent*’ (Sofia). As Reese described, ‘*I think having a group facilitator who did hear voices was a good thing because they could understand what you were saying and you could kind of tell them because they lived through it as well’*. While these participants were not opposed to having mental health professionals as facilitators, they did note that the facilitators would need to have HVM‐aligned values: ‘*the medical model… if there's a nurse [facilitating] for example…that's my only reservation*’ (Rita).

Over the course of the group, many members reported feeling a deep sense of connection to others and belonging to this ‘*mini community*’ (Reese), wherein hearing others' stories served as a source of ‘*inspiration*’ (Reese), ‘*strength*’ (Malcolm) and ‘*hope*’ (Rita). As Malcolm described, ‘*It was like a little family’* in which he felt ‘*accepted*’ for the first time and connecting with others helped him realise he was not alone: ‘*I always felt like I was alone in this journey. And then when I joined the group, I realised I'm not alone. There's other people out there as well. And it was like a comfort blanket knowing that’*.

### Seeking to learn

Many participants described a relational pattern that centred on learning information about voice hearing and were primarily focused on gaining new knowledge, predominantly in the form of coping skills, from the group. For these individuals, information from both facilitators and other members was equally valuable, given that they tended to approach voice hearing as a problem to be solved and, as such, looked for discrete skills that could bring them closer to living the lives they wanted to live. Individuals who came to the group primarily seeking to learn tended to be younger, from early intervention in psychosis or early detection and intervention services, and to have begun hearing voices relatively recently.

#### Searching for solutions

Participants who came to the group with the primary goal of learning more about voice hearing expressed a broad desire for ‘*knowledge*’ (Nigel) and ‘*understanding*’ (Roger) regarding their experiences. Some participants had specific aspirations, such as hearing voices ‘*less often*’ (Roger), helping voices to ‘*calm down*’ (Angela), or learning coping skills and ‘*techniques*’ (Angela). Roger expressed, *‘[I'd like] for them to be like not, not as harmful’*, and Nigel stated, ‘*I think I can maybe gain more, new knowledge about hearing voices…and like ways of cope‐ coping strategies’*. For this group, coping strategies were meant to mitigate voice‐related distress rather than to facilitate a change in sense‐of‐self or reduce a sense of isolation.

At the outset, these individuals placed less emphasis on wanting to share their experiences with others in order to be understood and validated and rather wished to hear others' stories in order to see if they could gain skills that may be particularly relevant to their current circumstances:

I haven't met anyone a similar age to me who hears them, so I feel like that's one of the main reasons I want to do it. To hear people's experiences my own age. I might get, um, different coping techniques or just ways they deal with stuff. (Tyler)

For these participants, hesitation around coming to the group tended to centre on meeting new people and learning group norms. As Roger stated, ‘*I weren't [sic] really sure about whether I was gonna do it or not…just like all the people…cos I'm not really a people person’*. Likewise, Angela felt that: ‘*I'm overwhelmed coming to the group…I just don't know what to expect’*.

#### Cameras enabled the modulation of engagement but inhibited group ownership

Participants who were primarily seeking to learn tended to have their cameras off, though did not feel like this was a barrier to their participation: ‘*Still we can share ideas and things like that. We share our different perspectives or ourselves or what we want to do or what we want to discuss*’ (Angela). This decision could be influenced by mood and/or feelings of social anxiety; for example, as stated by Roger, ‘*sometimes I literally just woke up and looked a mess. And like other times I was just anxious and didn't want to put [the camera] on’*. For participants like Roger, the expectation was that the group could accommodate him and his needs, rather than him changing to accommodate the group, thus potentially reflecting a diminished sense of collective group ownership. However, while Roger also stated he ‘*didn't really care that much*’ that other people could see him during the few occasions he did turn his camera on, other participants like Tyler indicated that being seen was deeply vulnerable, as he was no longer able to shield himself behind a screen and therefore opened himself up to facing rejection:

I think it's the thought of having a face to the [name]. It's probably the scariest part of it…it's probably the scariest part of it where you don't want people, just don't want people to put your experience to a face…you want it to stay a secret. Um, especially when you've never spoken to these people before. It's a lot harder. Probably the reason why some people stuck with the habit of just keeping it off. Cos they just never felt that confidence. Or they never felt willing to put a face to the [name].

Other participants likewise cited that shame was responsible for their reticence in turning on their camera: ‘*I'm embarrassed. That was the reason why I'm turning [it] off*’ (Angela).

Other members felt that a lack of camera enhanced their participation because it enabled them to modulate their engagement and thus, in turn, support them to open up about their voice hearing experiences: ‘*I think it allowed people to speak more freely*’ (Tyler). By extension, these individuals felt more comfortable being vulnerable online because the physical distance afforded by the online medium acted as a shield between them and the other participants, ultimately enabling them to retreat into a safe territory if needed: ‘*I'm a bit more, way more relaxed…I can mute my camera and be muted at times. I can't in a physical location…I could like say more when it was online*’ (Nigel).

While these participants still reported feeling more connected to individuals who had their cameras on, they were less inclined to express an explicit preference about what others did. In contrast, however, other participants did express concern about whether group members who didn't have their camera on were fully engaging with the group: ‘*If it's off, you can't tell if someone's engaging in the group properly*’ (Tyler). This made group members more reticent to share as they couldn't as easily discern whether the other members could be trusted.

#### A useful addition to care

Participants who attended the group to learn placed particular value on ‘*listening*’ (Nigel) to the stories of others. This not only included others' voice hearing experiences, but also their lives more generally, as it enabled them to realise that there was more to their identities than voice hearing alone, and that there was the potential for them to live full lives as someone who hears voices. This, in turn, was often a stark contrast to the messages they had received from mental health services. As Nigel described, ‘*It was useful to hear that…people my age and older can still hear voices…I think it was interesting to hear about…and what [Malcolm] said about his [name of hobby] group was interesting’*. However, these participants were also more hesitant to verbally contribute to the group, and likewise expressed doubt about whether their contributions were helpful to other members, thereby highlighting a challenge in establishing mutually responsible relationships. Some did not seek connections at all: ‘*I probably could [connect] but I didn't really want to*’ (Roger), whereas others felt a connection would build over time, but this was nevertheless not of central importance: ‘*I don't think I was in the group long enough to build the proper connection. But hearing other people's stories and how they deal with things did help*’ (Tyler).

For participants seeking to learn, the group represented an important addition to their care, rather than an alternative form of support with distinct norms and values. As such, they evaluated the utility of the group based on how much ‘*interesting*’ (Nigel) or useful information they could gain from it. Accordingly, these participants placed less importance on having a lived‐experience facilitator; rather, they emphasised that the ‘*skills*’ (Nigel) of the facilitator were paramount, regardless of their background. Participants likewise emphasised that even without the lived experience, professional facilitators could ‘*ask relevant questions and try to get a deeper understanding and meaning out of your answer*’ (Tyler). For individuals in this group, the role of the facilitator was one of asking exploratory questions and ensuring that the conversation continued to flow. Participants did not feel the same sense of group ownership as those seeking to connect, and therefore relied on the facilitators to hold the group space. This, in turn, enabled them to modulate their engagement; if they didn't feel comfortable or confident to carry the conversation, they could rely on the facilitators to support the group.

### Voices' experiences in the group

Participants reported that their voices had several different responses to being in the group, although these were not clearly delineated based on whether the participant was primarily seeking to connect or seeking to learn. Many voices ‘*felt threatened*’ (Roger) at the beginning of the group because ‘*it's all about them*’ (Roger) wherein ‘*the more we got into it and the more started discussing, they started to get a bit more agitated’*. (Tyler). For Tyler, this initial apprehension from his voices was a ‘*normal*’ experience that he was not bothered by, while Roger expressed that although ‘*they hated it at first, they actually started to like it toward the end*’. He noted that this was likely because his voices learned that the other members accepted and were not trying to get rid of them. As a result, the voices themselves felt as if they could express themselves more freely, be understood by the other group participants, and ultimately start to get their own needs met.

However, other participants' voices struggled more significantly in the group. Despite Reese and Malcolm feeling accepted themselves, their voices would nevertheless criticise their contributions both during and after the group, which resulted in a sense of fear around other members and apprehension about continuing to attend:

[During the break in the middle of the group] that's when the voices were worse… ‘don't go back on it,’ ‘they hate you’…the voices going through my head. And I [found] that a little bit of a struggle coming back onto the meeting…and I think the voices felt a bit scared (Malcolm).

Reese reported similar experiences, but likewise felt a sense of camaraderie from the fact that he was not the only one who experienced backlash from his voices: ‘*Another good thing about the group was that you'd see people, you'd think ‘oh yeah they're doing some good things’ but then they said they struggled with the voices afterward. Like, it's not just me then*’. By sharing the experience of hearing critical voices in the group, both Malcolm and Reese were able to address this feeling of alienation, realise that their voices' comments directly reflected their own self‐esteem and fear of rejection, and therefore ultimately continue in the group.

Sofia, in contrast, felt her voices ‘*enjoyed*’ the group, reporting ‘*they used to calm down when I had the group’*. She speculated that this may have been because both she and the voices felt accepted in the group, and because she was able to discern a clear function to the voices' threatening comments. Malcolm, despite hearing criticism from distressing voices, cited that a positive voice which he previously seldom heard became more prominent over the course of the group: ‘*and then this good voice, which I don't hear very often, it'll come out and then I'll hone in on it and think well yeah, I'm gonna have to [come to the group]…I heard it more once I'm in the group*’.

### Impact of the group

#### Impact on voices

Many participants reported that the group had helped them understand, relate and cope with their voices in more constructive ways. Participants ‘*picked up a couple of coping strategies*’ (Tyler) and ‘*learned techniques*’ (Angela) from other members, and even participants who were not inclined to try to connect with one another were nevertheless able to learn valuable information from being in the group. For Roger, this process arose both from listening to others as well as being asked specific questions about the phenomenology of his voice hearing experiences and how he responded to them: ‘*it's just like understanding…so I work with [the voices] more now and actually know what to do…what's more likely to help’*.

Participants like Tyler also learned specific skills in responding to his voices, ‘*some of the experiences you had yourself made me realise I can just say ‘no’ to [the voices]. I don't have to sit there and take it. I can literally say ‘no’ to them’*. Similarly, Reese learned about engaging with his in a more peaceful way:

We found in the group people engage with [their voices]…we were never told [by services] to engage with our voices. Just to shout at it and tell it to shut up which would make it worse…other people engage with their voices and actually speak to them and try to reason with them…that gave me like a new perspective on how to cope with the voices.

In some case, learning new skills also resulted in a sense of empowerment. For Sofia, this resulted in being less ‘*scared*’ of her voices, whereas Roger described how, ‘*I think [the voices are] the same but…I'm in a better position to help myself’*.

#### Impact on sense of self and Hope for the future

By hearing and connecting with the stories of others, participants reported a new level of self‐acceptance. This often resulted from having their experiences normalised: ‘*I think for the first time, just accepting that I hear voices and stuff. It's just normal for a lot of people and not just meself [sic] really*’ (Reese). Participants likewise felt more accepting of their voices, as described by Rita:

My eyes have been opened…I feel more confident about my voices, I feel less stigmatised. And I feel just generally more accepting of my voices. Which I didn't have before. It was always a battle. It was always like they shouldn't be there. And you know, ‘we've got to subdue them’…but I'm more accepting of them.

As a result, participants reported feeling more ‘*open*’ (Nigel) to confide in others outside the group about their voices. Sofia felt she was ‘*more brave to talk to [her] friends*’ about her experiences, and Rita ‘*told people since [she's] joined the group’*, which she reported to be ‘*quite liberating’*. Participants reported taking more chances as well, including ‘*going out more*’ (Angela) and ‘*joining other groups*’ (Sofia). Malcolm summarised the change in his life as, ‘*I find myself being a bit more braver’*.

Beyond these tangible changes, participants also reported that attending the group fundamentally shifted what they thought was possible for their future. Seeing other group members live full and fulfilling lives instilled some participants with a sense of agency and confidence in dealing with setbacks. As Nigel described, ‘*I think I can achieve things in the future. And I sort of thought that wasn't possible before’*. Likewise, while Rita stated, ‘*I feel more hopeful and more like I can go on functioning in my life with my voices*’ Reese felt that, ‘*if someone can do that, then I'll hope. Who knows, in the future, maybe I can do something like that as well’*. Malcolm similarly reported feeling like a more active player in his life, ‘*I'm just going to grab my opportunities now*’ whereas Sofia felt a new‐found sense of hope for her future, ‘*I started looking forward to my life now. To see what's next’*.

## DISCUSSION

This study provides the first account of NHS service users' experiences of online HVGs and expands on previous research on HVGs in statutory mental health settings (Kalofonos et al., 2023) by examining how the context of the group shaped participants' relationships within it, as well as investigating both voices' and participants' impressions of the group more broadly. There were two relational orientation tendencies reported by group participants: those seeking to connect and those seeking to learn, with the former viewing the group as a place to actively engage with others with a goal of reducing shame and isolation, and the latter primarily focused on accumulating knowledge that may ameliorate distressing voice hearing experiences. Duration of voice hearing and service use seemed to play a key role in this, with those with a longer duration of both voice hearing and service use expressing a stronger desire to connect with others, and those with a shorter duration of voice hearing and service use primarily seeking to gain new information.

In this respect, these styles of relating are somewhat consistent with Krasnova et al.'s (2015) construct of ‘information sharing’ versus ‘information consumption’ on social media, wherein the former reflects use that is active in nature, including sharing stories and interacting with others' posts (e.g., through commenting or ‘liking’), while the latter entails passively browsing content shared by others. While this construct was limited to social media use, there are some notable similarities in the present study, indicating that these modes of engagement may exist in online communities beyond those on social media. It must be noted, however, that this binary categorisation was too restrictive for the present study, where participants noted how features such as camera use could enable a building of trust over time, which ultimately may support a movement from passive to active engagement.

These findings further highlight some of the potential challenges implementing peer values into clinical services. While a subset of participants welcomed an alternative form of support, the remainder of participants viewed the group as simply another addition to their care: an intervention in which there was information to consume, as opposed to a group in which to actively participate. This is consistent with previous research highlighting that structural constraints in statutory services may inhibit the establishment of mutually responsible relationships (Ogundipe et al., 2019). However, despite these challenges, participants nevertheless reported positive impacts of group participation, including learning new coping skills, enhanced understanding of voices, reduced isolation and improved hope for the future. While these latter findings are consistent with those of Corentin et al. (2023), it is perhaps notable that of the four distinct benefits of HVGs outlined by Hornstein et al. (2024), only one was reported by the participants in this study: establishing camaraderie. This is perhaps a consequence of the NHS context and the fact that for many participants, exploring their experiences in depth was of less importance than learning new coping skills and connecting with other voice hearers. However, in line with Hornstein et al. (2021), this study highlighted that participants in NHS‐based groups can still attend the HVG for a variety of disparate reasons and a single group can still meet these diverse needs. Hornstein et al. (2020) likewise posit that the phenomenology of, and meaning assigned to, an individual's voice hearing experience will impact their reasons for attending and experiences within the group. While data in the present study were not sufficient to investigate this supposition, future research may benefit from this specific line of inquiry, especially given that the phenomenology and appraisal of voice hearing experiences may be particularly distressing for those requiring support from clinical services.

These findings likewise have noteworthy implications for online forms of support. Norms and expectation around camera use played a significant role in contributing to participants' orientation tendencies in the group, with those seeking to connect tending to expect universal camera use and those seeking to learn preferring to remain invisible. This disparity in preference was a further challenge to establishing mutually responsible relationships within the group as participants reported that some group members felt ownership over the space whilst others did not. This finding is perhaps unsurprising given the growing body of literature stating that participants having cameras off is a barrier to group cohesion for both participants and facilitators alike (Dreisoerner et al., 2023; Havlik et al., 2023). Facilitators of future groups should endeavour to have transparent conversations within groups about expectations surrounding camera use in order to support participants to both exercise self‐determination while remaining invested in the other group members.

This is also the first study to report on the diverse, dynamic experiences that voices themselves may have within HVGs, and it is important to note that even those participants whose voices were critical, and at times adversarial, during the group still reported benefits from participation. Future group facilitators should be mindful of the fact that voices may have their own opinions of and experiences in the HVG, and that this may be a productive avenue for discussion. Attention should also be given to exploring why voices may be responding in a particular way and highlighting how certain responses (e.g., a voice being critical of a participant's contribution) may in fact be a source of commonality between group members.

While this study took steps to maximise trustworthiness, it must also be viewed considering its limitations. AB acted both as group facilitator and interviewer, and while this may have enabled participants to speak more openly due to established rapport, it is likewise possible that this dissuaded disclosure of negative experiences within the group. Future studies may therefore endeavour to conduct interviews with an individual who was independent from group facilitation.

A longitudinal qualitative analysis was planned for this investigation. However, due to limited data from baseline interviews, it was not possible to compare domains such as voice phenomenology, voice appraisal, or sense of self as a voice hearer between baseline and end‐of‐study. The analysis would likewise be further nuanced by including the participant who completed the baseline but end‐of‐study interview, as her perspective of the group would likely be different from those who continued to engage with the group throughout the duration of the study. Further, no responses were received during efforts to member check findings with study participants, and future studies would undoubtedly be strengthened by incorporating participant feedback into the analysis.

In conclusion, this study provides new evidence for how an online HVG in the NHS could be experienced as an acceptable and beneficial form of support. Two relational orientation tendencies were reported, with some participants seeking to connect and others seeking to learn. The online medium, particularly as it pertained to camera use, further shaped their perspectives of the relational dynamics within the group. Nevertheless, regardless of orientation tendency, participants noted substantial benefits from group participation. Future research should now endeavour to explore the efficacy of such interventions in an NHS context and address the inevitable challenges of potentially implementing an ideologically distinct approach into the NHS.

## AUTHOR CONTRIBUTIONS

**Alison Branitsky:** Conceptualization; investigation; funding acquisition; writing – original draft; methodology; writing – review and editing; formal analysis; project administration; data curation. **Samantha Bowe:** Investigation; writing – review and editing; supervision; formal analysis. **Sandra Bucci:** Conceptualization; methodology; writing – review and editing; formal analysis; supervision. **Anthony P. Morrison:** Conceptualization; writing – review and editing; methodology; formal analysis; supervision. **Eleanor Longden:** Conceptualization; writing – review and editing; methodology; formal analysis; supervision. **Lee D. Mulligan:** Investigation; writing – review and editing; formal analysis; supervision. **Filippo Varese:** Conceptualization; writing – review and editing; methodology; formal analysis; supervision; project administration.

## FUNDING INFORMATION

This study is funded by a President's Doctoral Scholarship awarded to AB by the University of Manchester. SBu, AMP, LDM and FV are supported by the NIHR Manchester Biomedical Research Centre (NIHR 203308). SBu is also supported by the National Institute for Health and Care Research research professorship (NIHR 300794). The views expressed are those of the author(s) and not necessarily those of the NIHR or the Department of Health and Social Care.

## CONFLICT OF INTEREST STATEMENT

AB is a trustee of the English Hearing Voices Network. Her tenure as a trustee began after the trial procedures were complete. AB and EL provide paid training and consultancy on the Hearing Voices Movement. SBu is a director and shareholder of CareLoop Health Ltd, which develops and markets digital therapeutics for schizophrenia and a digital screening app for postnatal depression. SBu also reports research funding from the Wellcome Trust and NIHR. EL reports research funding from the NIHR. The rest of the authors declare no conflict of interest in relation to this research.

## ROLE OF THE SPONSOR

The sponsor has no direct role in the development of the protocol or delivery of the research procedures. The views expressed are those of the authors and not necessarily those of the University of Manchester.

## Supporting information


**Data S1.** Pre‐group topic guide.

**Data S2.** Post‐group topic guide.

## Data Availability

Research data are not shared.
