# Supplementary material for: ‘It was like a little family’: A qualitative study of online hearing voices peer support groups in the NHS
Source: Psychol Psychother. 2026 Apr 12;99(3):995–1012. doi: 10.1111/papt.70064 (PMC13456475; doi:10.1111/papt.70064)
Supplement: Supplementary file 1 [file PAPT-99-995-s001.docx]

Supplementary Materials 1. Pre-Group Topic Guide

Part 1. Online Experiences

1. Could you tell me a bit about any experiences you’ve had with online groups or meetings?
   a. Prompt: support groups, telehealth meetings
   b. Prompt: how they found being in the group
   c. Prompt: advantages/disadvantages, likes/dislikes
2. Do you have any thoughts about this group meeting online as opposed to face-to-face?
   a. Prompt: potential benefits/challenges
3. As you know, this group is going to meet online. Is there anything about an online group that is particularly appealing?
4. What concerns do you have about meeting online?

Part 2. Voice Hearing Experiences

5. Could you tell me a bit about your voice hearing experiences?
   a. Prompt: when did you start hearing them?
   b. Prompt: have they changed over the course of your life?
   c. Prompt: what kinds of things do they say/what do they sound like?
6. Do you have any ideas about why you started hearing voices?
7. Do you have any ideas about what it means to be somebody who hears voices?
   a. Clarification: for example, some people associate voice hearing with having a mental illness. What are your thoughts about this?
   b. Prompt: how do you feel about these explanations?
   c. Prompt: are there any other explanations that you prefer?
8. When you first started hearing voices, did you tell anybody else? How did they respond?
   a. Prompt: what was that experience like?
   b. Prompt: how would you have liked people to have responded?
9. Is there anybody you talk to now about your voices? What is that like?
10. How does hearing voices make you feel about yourself?
11. We know that many people who hear voices have experienced a range of adverse life experiences. We think that it is important to acknowledge this. As a matter of routine, we ask everybody if there are any life experiences you feel are connected to your voice hearing experiences that you’d like to mention.

Part 3. Expectations of the Group

12. What do you hope to get out of attending the hearing voices group?
13. What concerns do you have about attending the hearing voices group?
14. Do your voices have any thoughts or opinions about attending the hearing voices group?
