# Supplementary material for: ‘It was like a little family’: A qualitative study of online hearing voices peer support groups in the NHS
Source: Psychol Psychother. 2026 Apr 12;99(3):995–1012. doi: 10.1111/papt.70064 (PMC13456475; doi:10.1111/papt.70064)
Supplement: Supplementary file 2 [file PAPT-99-995-s002.docx]

Supplementary Materials 2. Post-Group Topic Guide

Part 1. Joining the group and group structure

1. Could you tell me what you remember about deciding whether or not to take part in the HVG?
2. Broadly speaking, how did you find the structure of the group?
   a. Prompt: feedback on group length
   b. Prompt: feedback on ability to join early
   c. Prompt: feedback on break and timing of break
   d. Prompt: feedback on structure of group (e.g., show-and-tell, inviting agenda items)
   e. Prompt: feedback on 6-month timeframe
3. Thinking back to the beginning of the group, having cameras on and deciding whether or not to be on video was a big question for people. Can you remember why you wanted to have your camera on/off at the start of the group?
   a. Prompt: (if decision changed) what prompted you to change your mind?
   b. Prompt: how did you find being able to see other group members?
   c. Prompt: how did you find that other group members could see you?
   d. Prompt: could you connect to people differently depending on whether they had their cameras on?
4. What was the impact of the group being online as opposed to face-to-face?
   a. Prompt: how was engagement impacted?
   b. Prompt: any limitations
   c. Prompt: perceived differences from face-to-face
5. What is your online life like outside the group?

Part 2. Experiences in the group

6. How did you find being part of the group?
7. How did your voices find being part of the group?
8. Were there any parts of the group that were particularly helpful?
9. Were there any parts of the group that were particularly unhelpful?
10. Can you tell me about your experience of connecting to other group members online?
    a. Prompt: was there a connection?
    b. Prompt: what enabled the connection?
    c. Prompt: what did you learn from the other group members?
    d. Prompt: what do you feel like you contributed to other group members?
11. What kinds of experiences or information was particularly useful to hear?
12. In the group, we had people from different ages and services (EIPs and CMHTs). How did you find that?
13. How did you find the facilitation of the group?
    a. Prompt: impact of both peer and professional facilitators
    b. Prompt: was there anything the facilitators did that was particularly helpful/unhelpful
14. Did you feel the group was different from sources of support you have been offered in the past?

Part 3. Impact of the group

15. What, if anything, did you get out of group participation?
16. What impact, if any, did the group have on your voices?
17. What impact, if any, did the group have on how you feel about yourself?
18. What impact, if any, did the group have on how you understand your voices?
19. What impact, if any, did the group have on how you relate to other people?
20. What impact, if any, did the group have on how you use or interact with mental health services?
21. What do you think will be different or you now that the group is over?
22. How has the group impacted how you think about your future?
23. What were the downsides in taking part in the group?
24. What, if anything, would you change about the group?
25. How are things now compared with before you started the group?
26. Do you think these groups should be made more widely available?
    a. Prompt: in the NHS? In the community?
    b. Prompt: what should they look like?
